# Repeated Assessments of Informed Consent Comprehension among HIV-Infected Participants of a Three-Year Clinical Trial in Botswana

**DOI:** 10.1371/journal.pone.0022696

**Published:** 2011-10-27

**Authors:** Lelia H. Chaisson, Nancy E. Kass, Bafanana Chengeta, Unami Mathebula, Taraz Samandari

**Affiliations:** 1 Berman Institute of Bioethics, Johns Hopkins University, Baltimore, Maryland, United States of America; 2 Department of Health Policy and Management, Johns Hopkins Bloomberg School of Public Health, Baltimore, Maryland, United States of America; 3 Botswana-USA Partnership, Gaborone and Francistown, Botswana; 4 Division of Tuberculosis Elimination, Centers for Disease Control and Prevention, Atlanta, Georgia, United States of America; University of Cape Town, South Africa

## Abstract

**Background:**

Informed consent (IC) has been an international standard for decades for the ethical conduct of clinical trials. Yet frequently study participants have incomplete understanding of key issues, a problem exacerbated by language barriers or lack of familiarity with research concepts. Few investigators measure participant comprehension of IC, while even fewer conduct interim assessments once a trial is underway.

**Methods and Findings:**

We assessed comprehension of IC using a 20-question true/false quiz administered in 6-month intervals in the context of a placebo-controlled, randomized trial for the prevention of tuberculosis among HIV-infected adults in Botswana (2004–2009). Quizzes were offered in both Setswana and English. To enroll in the TB trial, participants were required to have ≥16/20 correct responses. We examined concepts understood and the degree to which understanding changed over three-years. We analyzed 5,555 quizzes from 1,835 participants. The participants' highest education levels were: 28% primary, 59% secondary, 9% tertiary and 7% no formal education. Eighty percent of participants passed the enrollment quiz (Quiz1) on their first attempt and the remainder passed on their second attempt. Those having higher than primary education and those who took the quiz in English were more likely to receive a passing score on their first attempt (adjusted odds ratios and 95% confidence intervals, 3.1 (2.4–4.0) and 1.5 (1.2, 1.9), respectively). The trial's purpose or procedures were understood by 90–100% of participants, while 44–77% understood randomization, placebos, or risks. Participants who failed Quiz1 on their initial attempt were more likely to fail quizzes later in the trial. Pass rates improved with quiz re-administration in subsequent years.

**Conclusions:**

Administration of a comprehension quiz at enrollment and during follow-up was feasible in a large, international collaboration and efficiently determined IC comprehension by trial participants. Strategies to improve understanding of concepts like placebos and randomization are needed. Comprehension assessments throughout a study may reinforce key concepts.

## Introduction

### Background

The protection of human subjects in medical research is a fundamental concern of bioethics. Informed consent is one means of providing such protection. Through the delivery of key study information, informed consent allows potential participants, based on their own values, to decide which risks, benefits, and procedures are acceptable to them. While informed consent is expected to be a dialogue between researcher and potential participant, consent information is typically provided through a written consent form as well, which is also used to document the participant's agreement to enroll in the study.

While informed consent documents are useful as a means of standardizing information, research participants in multiple settings and in multiple studies have been shown to have incomplete or inaccurate understanding of many facets of information provided through the informed consent process [1]–[5]. Unfamiliar terms such as randomization and placebo are particularly hard to understand [6]–[9], as are concepts related to risks [10], [11], benefits [12]–[14], and the freedom to withdraw from the study [15]–[18]. Additionally it has been shown that many trial participants are unaware that they are enrolled in research [18]–[20]. Incomplete and/or inaccurate understanding has been attributed to several factors, including unfamiliarity with research, low levels of education, hopes for clinical benefit, and problems with consent forms being too lengthy or complex [5], [21]–[24]. While such problems exist in both developed and developing country contexts, they may be amplified in international settings where language barriers or lack of familiarity with research itself may make understanding of research concepts harder to achieve [3], [25]–[27].

Further hampering research understanding is the decrease in the ability to remember study information over time [28]–[31]. As such, the Council for International Organizations of Medical Sciences (CIOMS) international ethical guidelines for research emphasizes the continuous nature of informed consent [32]. It is unclear whether the investigators of longitudinal studies review important information with trial participants after studies have begun [29], and we are not aware of any regulations or guidelines that formally require this in longitudinal projects [29], [33]. The difficulty of ensuring adequate comprehension of study information has led some investigators to recommend that participants' understanding be assessed after consent discussions have occurred, but prior to enrollment [11], [34]–[36]. While researchers report believing that testing understanding is important, limited evidence suggests that only a minority of researchers actually engage formally in an assessment process [37]–[40]. A small number of investigators have published findings from attempts to measure participants' understanding prior to enrollment, with an even smaller number having conducted interim assessments once research is underway [36], [41], [42].

A clinical trial conducted in Botswana from 2004–2009 provided an opportunity to examine how well participants understood study information when first told about the study, and to measure their understanding over time. The Isoniazid Prevention Therapy (IPT) trial, part of an ongoing collaboration between the U.S. Centers for Disease Control and Prevention (CDC) and the Botswana Ministry of Health, was a randomized, double-blind, placebo-controlled study of limited (6 months) vs. continuous (36 months) IPT among HIV-infected persons ≥18 years of age. This three-year study allowed investigators to detect any differences in rates of active TB incidence between the two study arms. Participation involved monthly clinic visits over the 36 months. The informed consent form for the trial was first developed in English by study investigators, translated into Setswana, and back-translated into English to ensure consistency of meaning.

In an effort to ensure that participants adequately understood study information, investigators developed a 20 item true/false comprehension quiz to administer following the informed consent discussion, but prior to enrollment. To be able to join the study, participants had to be able to answer at least 16 of the 20 items correctly on the quiz. Potential participants who failed further discussed the protocol with study nurses, and were then allowed to repeat the quiz. Those who failed the quiz twice were not allowed to enroll in the clinical trial.

This manuscript reports on the analysis of the comprehension quiz data. Specifically, we examined the extent to which participants obtained a passing quiz score at enrollment, factors associated with passing or failing scores, likelihood of retaining study information over time, and which questions were most likely to be answered correctly and incorrectly.

## Methods

### IPT Trial

Recruitment for the IPT trial took place from 2004 through 2006. In December 2006, the investigators amended the study protocol such that the comprehension test was re-administered to study subjects every 6 months throughout the remainder of their time in the trial, allowing investigators to measure participants' understanding of the study over time. Participants were not removed from the study at this stage if a passing score (≥16/20 correct responses) was not obtained, but nurses reviewed relevant information with participants, allowing reinforcement of information that had been either forgotten or misunderstood. As the decision to re-administer the quiz every 6 months was made 2 years after enrollment began, participants who enrolled in 2004 received their first follow-up quiz about 2 years into the study (and subsequently at months 30 and 36). Subjects who first enrolled in 2006 were re-quizzed every 6 months after enrollment, for a total of up to 6 quizzes.

### Data collection

Data for this study consisted of all available enrollment and follow-up quizzes from participants in the IPT trial. Data were abstracted from the hard copy paper quizzes in July 2008 from the 2 study sites in Gaborone and Francistown, Botswana, and were entered into a Microsoft Access database. Original paper quizzes had no participant-identifying information and had been labeled with a participant identification number (ID) and housed in locked warehouses. Data abstracted included variables present on the comprehension quiz: participant ID; day, month, and year that the quiz was administered; answers the participant gave for each of the 20 true/false quiz items; and language of administration (English or Setswana).

Quality assurance was carried out for the quizzes from a randomly selected 12% of the participant IDs following data collection. The entry of their quizzes into the Access database was checked for accuracy against the original paper quizzes and inconsistencies were corrected.

Electronic data files were transported to the Johns Hopkins Berman Institute of Bioethics for analysis. The Access database was converted to a STATA file using Stat Transfer. Data were analyzed using STATA 10.

Additional data, including demographic variables of study participants, were obtained from the study team and linked with quiz data. Subjects whose enrollment date did not match the date of the enrollment quiz in the original dataset within a window of 30 days were eliminated from the dataset.

### Variables

A quiz number (1–6) was assigned to the quizzes for each participant ID, with the enrollment quiz always being quiz 1. An indicator was created for participants who had 2 baseline quizzes, as this signified that the participant was reported to have failed the first enrollment quiz and was therefore given the enrollment quiz twice. An indicator was created for each question so that we could identify which questions were answered correctly, incorrectly, or left blank, and an additional variable was created to sum the number of questions answered correctly. Variables were created to determine the time since the enrollment quiz and the time since the previous quiz.

To ensure accurate interpretation of quiz items we asked 8 individuals who were unrelated to the trial and who were fluent in both English and Setswana to independently review and back-translate 4 of the Setswana quiz questions that were suspected to have been incorrectly translated into Setswana after the quiz analysis began.

### Analysis

Bivariate and adjusted logistic regression analyses were performed for the enrollment quizzes to determine associations between demographic features and rates of passing at enrollment, as well as the likelihood of answering specific questions correctly at enrollment. These logistic regression analyses were also performed for the entire longitudinal dataset.

Bivariate analyses were performed to determine the odds of passing each follow up quiz compared with the enrollment quiz and compared with the previous quiz, as well as the odds of passing a quiz by the interval since the last quiz.

### Ethical Review

The Botswana IPT Trial was approved by the Botswana Ministry of Health ethics committee and the CDC institutional review board. This secondary analysis of comprehension quiz data was approved by the Johns Hopkins Bloomberg School of Public Health IRB and the Botswana ethics committee and CDC institutional review board. The trial was registered at clinicaltrials.gov (NCT00164281).

## Results

### Data Included

From 2004 to 2006, 4,018 individuals agreed to screening for the IPT trial. Of 1,860 individuals deemed ineligible for participation, 25 were ineligible because they failed the comprehension quiz twice. A total of 1,995 participants were enrolled in the IPT trial [43]. Of the 1,995 IPT trial participants, 1,971 had at least one quiz available for data entry; a total of 5,882 baseline and follow-up quizzes were entered into the database. The quizzes of 136 participants were excluded from analysis because their enrollment dates did not match those in the study database, and 163 quizzes were excluded because they were not dated. Additionally, during the 3-year period of observation, 176, 77, 46 and 11 participants voluntarily withdrew from the study, died, attended study visits less than every 6 months on average or were lost to follow-up, respectively. The final dataset contained 5,555 quizzes from 1,835 participant IDs. Of these, 33% had only an enrollment quiz, while less than 2% of participants had more than 5 total quizzes (Table 1).

**Table 1:** Number of comprehension quizzes completed by HIV-infected adults enrolled in a tuberculosis prevention trial, Botswana 2004–2008.

	Freq.	%
Enrollment	1,835	33
2^nd^ attempt, enrollment	96	2
Quiz 2	1,436	26
Quiz 3	1,306	24
Quiz 4	853	15
Quiz 5	28	<1
Quiz 6	1	<1
**Total**	**5,555**	**100**

Comprehension quizzes were administered at screening for enrolment (Quiz 1) and then every 6 months for a period of 36 months. However because not all participants were administered all quizzes, “Quiz 2” – for instance – refers to the second quiz taken regardless of the time elapsed since Quiz 1.

### Quality assurance

A total of 495 randomly selected quizzes for 233 participants were checked against the original paper quizzes. Six data entry errors were detected, resulting in an error rate of less than 0.05%.

### Descriptive data

The demographic characteristics of the study population are displayed in Table 2. The study population was largely female (72%), the median age was 33 years, and 32% were unemployed. Sixty-five percent had received at least a secondary education, 28% had received no more than a primary education, 9% had received education beyond the secondary level and 8% had no formal education.

**Table 2:** Characteristics of participants of the isoniazid tuberculosis preventive therapy trial for whom comprehension quizzes were available.

Characteristic	N	%
**Sex**		
Female	1,318	72
Male	516	28
Missing	1	<1
**Age**		
<20	8	0.4
20–29	586	32
30–39	833	45
40–49	337	18
50–59	63	3
60–69	6	<1
70+	1	<1
Missing	1	<1
**Occupation**		
Student	33	2
Administrator, Professional	156	8
Clerk, Technician	108	6
Service Worker, Sales	301	16
Crafts, Trades, Assemblers	107	6
Skilled Agricultural	5	0.3
Laborer, Miner, Construction, Transport	420	23
Unemployed	581	32
Other	122	7
Missing	2	<1
**Education**		
None	119	6
Primary	511	28
Secondary	1,033	56
Tertiary	164	9
Other	7	<1
Missing	1	<1

At enrollment, 47% of quizzes were taken in Setswana, although longitudinally 66% of all quizzes were taken in Setswana. Participants did not remain consistent in their choice of language for quiz administration; of the participants who had more than 1 quiz available for analysis, 10% took all of their quizzes in English and 37% took all of their quizzes in Setswana. More than half switched their quiz language preference throughout the trial.

### Comprehension quiz scores

The final data set included enrollment quizzes from 1,835 participants. The overall median score at enrollment was 17 correct responses from among 20 true/false questions. At enrollment, 1,475 participants (80%) passed on the first attempt, and 342 (19%) failed initially but then passed on their second attempt (the remainder did not have a score recorded). The median score for those who failed on their first attempt was 15.

There was a decrease in the proportion of participants who received a passing score for quiz 2 (63%). The odds of passing quiz 2 were significantly lower compared to quiz 1 (OR = 0.4 [0.4, 0.5]). The proportion of participants passing the comprehension quizzes rose following quiz 2; however the odds of passing remained lower than those of passing quiz 1 for all except quiz 5, for which the odds was not statistically significantly different than quiz 1 (Table 3).

**Table 3:** Odds of passing comprehension quizzes 1–5 compared to quiz 1 & previous quiz; odds of passing comprehension quizzes 1–5 by time since last quiz (months).

	Quiz number				
	Quiz 1	Quiz 2	Quiz 3	Quiz 4	Quiz 5
	n = 1817	n = 1418	n = 1298	n = 848	n = 28
Proportion passing	80%	63%	72%	76%	86%
OR (95% CI) compared to Quiz 1		0.4 (0.4, 0.5)	0.6 (0.5, 0.7)	0.7 (0.6, 0.9)	1.4 (0.5, 4.0)
OR (95% CI) compared to previous quiz		0.4 (0.4, 0.5)	1.5 (1.2, 1.7)	1.2 (1.0, 1.5)	1.9 (0.6, 5.5)

	Months since last quiz				
	1–6	7–12	13–18	19–24	>24
	n = 166	n = 683	n = 440	n = 562	n = 1742
Proportion passing	69%	65%	61%	69%	75%
OR (95% CI) compared to 1–6 months		0.8 (0.6, 1.2)	0.7 (0.5, 1.1)	1.0 (0.7, 1.5)	1.3 (0.9, 1.9)

### Passing over time

The proportion and odds of passing a repeat comprehension quiz by time since the last quiz (months) are displayed in Table 3. Of the participants with a score recorded, the proportion of participants passing quizzes dropped from 69% when the quiz was administered 1–6 months following the previous quiz to 65% at 7–12 months and 61% at 13–18 months. The proportion rose at 19–24 months and greater than 24 months. The odds of passing compared to 1–6 months were not statistically significant at any of these times.

### Associations with Passing/Failing Comprehension Quizzes

Higher rates of passing were significantly associated with higher educational attainment. Table 4 shows the proportion of participants passing the enrollment quiz by highest level of education as well as the odds of passing compared with no formal education. Those with secondary or tertiary education were significantly more likely to pass than those with no formal education. Participants over the median age of 33 had a lower odds of passing the enrollment quiz compared with those age 33 and younger (OR = 0.7 [0.5, 0.8]). Passing was not associated with sex at enrollment (OR = 1.1 [0.8, 1.4]).

**Table 4:** Odds of passing quiz 1 by highest level of education; odds of taking quiz 1 in English by highest level of education.

	Highest level of education^1^			
	None	Primary	Secondary	Tertiary
	n = 117	n = 509	n = 1021	n = 162
Proportion passing at enrollment	73%	76%	83%	89%
OR (95% CI) compared to no education		1.2 (0.8, 1.9)	1.9 (1.2, 2.9)	3.0 (1.6, 5.7)
Proportion taking enrollment quiz in English^2^	43%	43%	54%	78%
OR (95% CI) taking quiz in English compared to no formal education		1.0 (0.7, 1.5)	1.6 (1.1, 2.4)	4.9 (2.9, 8.2)
OR (95% CI) taking quiz in English compared to previous level		1.0 (0.7, 1.5)	1.6 (1.3, 2.0)	3.0 (2.0, 4.5)

1 Level of education coded as “Other” not included.

2 Only records with score recorded included.

Participants with higher education were more likely to take the quizzes in English than those with lower education (Table 4). Moreover, participants who took the quiz in English were more likely to pass all quizzes than those who took the quiz in Setswana (Table 5). The adjusted odds ratio indicated that individuals who took the quiz in English were more likely to pass regardless of highest level of education. Those with greater than primary education were grouped together to conduct adjusted odds ratios. Moreover, those with higher levels of education were more likely to pass regardless of the language in which the quiz was taken (Table 5).

**Table 5:** Unadjusted and adjusted odds of passing quizzes 1–5 by language and education.

OR Passing (95% CI)		
	Unadjusted	Adjusted
**Quiz 1** (n = 1809)^1^		
English	3.3 (2.5, 4.2)	3.1 (2.4, 4.0)
>primary education	1.7 (1.4, 2.2)	1.5 (1.2, 1.9)
**Quiz 2** (n = 1412)		
English	1.5 (1.1, 1.9)	1.3 (1.0, 1.7)
>primary education	1.5 (1.2, 1.8)	1.4 (1.1, 1.7)
**Quiz 3** (n = 1293)		
English	1.9 (1.4, 2.6)	1.4 (1.0, 2.0)
>primary education	2.4 (1.9, 3.1)	2.2 (1.7, 2.9)
**Quiz 4** (n = 846)		
English	2.4 (1.6, 3.6)	1.4 (1.0, 2.0)
>primary education	2.8 (2.0, 3.8)	2.2 (1.7, 2.9)
**Quiz 5** (n = 22)		
English	∞	∞
>primary education	3 (0.3, 26.2)	2 (0.2, 17.9)

Odds ratios (OR) shown compare quiz results of those quizzes taken in English to those quizzes taken in Setswana and compare participants who had greater than primary education to those who had either only primary education or no formal education.

1 Only participants with a score, language, and level of education included; participants with a level of education coded as “other” not included.

Participants who failed quiz 1 on their initial attempt and then passed on their second attempt (n = 342), were more likely to fail quiz 2 (93/251, OR 0.4, 95%CI0.3–0.5), quiz 3 (81/226, OR 0.4, 95%CI0.3–0.6), and quiz 4 (42/136, OR 0.5, 95%CI0.4–0.8) than participants who passed quiz 1 on their first attempt.

### Translation Reassessment

The translation reassessment conducted in the analysis phase revealed discrepancies in how 4 of the 20 questions had been translated. Notably, the eight new translators unanimously agreed that question 9, “By taking the medicine I have a very small chance of having damage to my liver,” was originally translated into Setswana as “This medication will help protect my liver.” This error changed the correct answer from true to false for those who took the quiz in Setswana. Subsequently, all data presented in this manuscript were adjusted to take into account which quiz response should be counted as correct. Additionally, 5 of the 8 translators back-translated the phrase “sugar pill” in questions 2 and 18 as “diabetes pill.” As there was some disagreement among the translators, the data were not adjusted.

### Response to specific quiz questions

Table 6 shows the rates of correct and incorrect answers at enrollment for each question, as well as the odds of those answering the questions in English correctly compared to those answering in Setswana. Over 90% of participants responded correctly to questions that related to study purpose and procedures. Twenty-three to 56% of participants answered questions related to placebos/blinding, adherence, and risks incorrectly. While participants who took the quiz in English were more likely to answer many questions correctly than those who took the quiz in Setswana, notably, those who took the quiz in English were not more likely to answer the questions regarding placebos correctly more frequently than those who took the quiz in Setswana.

**Table 6:** Proportion of participants answering comprehension quiz 1 questions 1–20 correctly (English & Setswana); Odds of answering correctly if quiz taken in English.

Comprehension Question	Category	English (n = 969)			Setswana (n = 866)			OR (95% CI)
		Correct	Incorrect	Blank	Correct	Incorrect	Blank	
Q1 “This study is about preventing tuberculosis in people living with HIV/AIDS”	Purpose	97%	1%	2%	90%	10%	<1%	18.0 (7.9, 41.5)
Q2 “I will know whether I am taking a sugar pill”	Placebo, Blinding	64%	34%	3%	65%	35%	<1%	1.03 (0.85, 1.3)
Q3 “This study will last for 3 years”	Procedures	97%	1%	2%	96%	4%	0%	6.03 (2.5, 14.5)
Q4 “Ten teaspoons of blood will be taken from my arm”	Procedures	81%	16%	4%	66%	33%	1%	2.6 (2.08, 3.25)
Q5 “I will have a chest x-ray done”	Procedures	97%	<1%	2%	100%	<1%	0%	0.55 (0.1, 2.98)
Q6 “I must continue taking the medicine even if the medication makes me ill”	Procedures, Voluntariness	77%	19%	3%	69%	30%	1%	1.72 (1.39, 2.14)
Q7 “I will no longer receive study medication if I stop taking the medication”	Adherence	65%	30%	5%	44%	56%	1%	2.79 (2.3, 3.38)
Q8 “There is almost no chance that someone will find out I am HIV infected”	Risks	85%	12%	3%	94%	6%	0%	0.43 (0.31, 0.61)
Q9 “By taking the medicine I have a very small chance of having damage to my liver”	Risks	81%	16%	3%	45%	54%	1%	6.0 (4.83, 7.46)
Q10 “I will be seen by a doctor or nurse each time I return for a visit”	Procedures	96%	2%	2%	98%	2%	<1%	0.86 (0.46, 1.6)
Q11 “I will not receive compensation for transport costs to come to the clinic”	Compensation	89%	9%	2%	89%	11%	<1%	1.15 (0.85, 1.57)
Q12 “I can refuse to continue in the study at any time”	Voluntariness	77%	21%	2%	76%	24%	0%	1.12 (0.9, 1.39)
Q13 “I must give a urine sample at each visit”	Procedures	77%	20%	3%	86%	14%	<1%	0.61 (0.48, 0.79)
Q14 “My blood will be tested for possible liver damage by the study medication”	Procedures, Risks	89%	9%	2%	93%	7%	<1%	0.71 (0.51, 1.0)
Q15 “If I become ill with AIDS I will no longer be in the study”	Procedures	74%	23%	3%	77%	22%	<1%	0.96 (0.77, 1.19)
Q16 “The isoniazid may make me sick by making me feel nauseated”	Risks	90%	8%	2%	87%	13%	<1%	1.61 (1.18, 2.18)
Q17 “Currently the Botswana government recommends one year of isoniazid preventive treatment for people living with HIV/AIDS”	Background	87%	11%	2%	84%	16%	<1%	1.55 (1.18, 2.03)
Q18 “A placebo is a sugar pill”	Placebo	78%	19%	3%	82%	18%	<1%	0.89 (0.7, 1.13)
Q19 “If I think I am hurt by this study I should contact the police station in Gaborone”	Risks	94%	4%	3%	94%	6%	0%	1.66 (1.08, 2.54)
Q20 “This study will help people living with HIV/AIDS by finding out if longer preventive treatment with isoniazid will prevent TB”	Purpose	96%	1%	2%	98%	2%	0%	1.65 (0.84, 3.27)

Across the entire dataset, questions related to study procedures were most likely to be answered correctly, whereas questions related to randomization, placebos, adherence, or risks were most likely to be answered incorrectly (Table 7).

**Table 7:** Proportion of participants answering comprehension quiz questions 1–20 correctly (English & Setswana); Odds of answering correctly if quiz taken in English.

Comprehension Question	Category	English (n = 969)			Setswana (n = 866)			OR (95% CI)
		Correct	Incorrect	Blank	Correct	Incorrect	Blank	
Q1 “This study is about preventing tuberculosis in people living with HIV/AIDS”	Purpose	97%	1%	2%	85%	15%	<1%	21.81(13.01, 36.54)
Q2 “I will know whether I am taking a sugar pill”	Placebo, Blinding	68&	29%	3%	64%	36%	1%	1.28 (1.14, 1.46)
Q3 “This study will last for 3 years”	Procedures	97%	1%	2%	96%	4%	<1%	3.73 (2.3, 6.05)
Q4 “Ten teaspoons of blood will be taken from my arm”	Procedures	80%	17%	4%	71%	28%	1%	1.89 (1.64, 2.18)
Q5 “I will have a chest x-ray done”	Procedures	94%	3%	3%	97%	3%	<1%	0.91 (0.66, 1.26)
Q6 “I must continue taking the medicine even if the medication makes me ill”	Procedures, Voluntariness	80%	17%	3%	75%	24%	1%	1.53 (1.33, 1.76)
Q7 “I will no longer receive study medication if I stop taking the medication”	Adherence	57%	40%	4%	54%	46%	1%	1.21 (1.08, 1.36)
Q8 “There is almost no chance that someone will find out I am HIV infected”	Risks	82%	15%	3%	90%	9%	<1%	0.56 (0.48, 0.67)
Q9 “By taking the medicine I have a very small chance of having damage to my liver”	Risks	73%	25%	3%	46%	53%	1%	3.45 (3.05, 3.91)
Q10 “I will be seen by a doctor or nurse each time I return for a visit”	Procedures	95%	3%	2%	97%	3%	<1%	0.95 (0.69, 1.33)
Q11 “I will not receive compensation for transport costs to come to the clinic”	Compensation	90%	8%	2%	91%	8%	1%	0.99 (0.8, 1.21)
Q12 “I can refuse to continue in the study at any time”	Voluntariness	75%	23%	2%	74%	25%	<1%	1.14 (0.99, 1.29)
Q13 “I must give a urine sample at each visit”	Procedures	85%	12%	3%	92%	8%	<1%	0.58 (0.49, 0.7)
Q14 “My blood will be tested for possible liver damage by the study medication”	Procedures, Risks	91%	7%	2%	95%	4%	<1%	0.58 (0.46, 0.74)
Q15 “If I become ill with AIDS I will no longer be in the study”	Procedures	80%	17%	3%	87%	12%	<1%	0.69 (0.59, 0.8)
Q16 “The isoniazid may make me sick by making me feel nauseated”	Risks	77%	21%	3%	79%	21%	<1%	0.99 (0.87, 1.14)
Q17 “Currently the Botswana government recommends one year of isoniazid preventive treatment for people living with HIV/AIDS”	Background	84%	14%	2%	82%	18%	<1%	1.34 (1.15, 1.57)
Q18 “A placebo is a sugar pill”	Placebo	75%	22%	3%	66%	32%	1%	1.65 (1.45, 1.88)
Q19 “If I think I am hurt by this study I should contact the police station in Gaborone”	Risks	94%	3%	3%	93%	7%	<1%	2.25 (1.68, 3.0)
Q20 “This study will help people living with HIV/AIDS by finding out if longer preventive treatment with isoniazid will prevent TB”	Purpose	96%	2%	2%	93%	7%	<1%	4.46 (3.06, 6.51)

### Likelihood of answering specific quiz questions correctly at follow-up compared to enrollment

Table 8 shows the odds of answering each question correctly compared with the enrollment quiz for quizzes 2–5. Participants had greater odds of answering correctly questions related to compensation (Q11), procedural questions related to providing a urine sample and having a blood test (Q13, Q14), and the effect that becoming ill with AIDS would have on participation (Q15). Participants had lower odds of answering correctly questions related to purpose (Q1, Q20), the procedural question related to having an x-ray (Q5), questions related to risks of someone finding out that the participant was HIV+ (Q8), having damage to the liver (Q9), and the possibility of becoming nauseated (Q16). Least likely to be correct was the question related to placebos (Q18).

**Table 8:** Odds of answering comprehension quiz questions 1–20 correctly at Quizzes 2–5 compared to enrollment quiz.

Comprehension Question	Quiz 2 OR (95% CI) (n = 1417)	Quiz 3 OR (95% CI) (n = 1298)	Quiz 4 OR (95% CI) (n = 848)	Quiz 5 OR (95% CI) (n = 28)
Q1 “This study is about preventing tuberculosis in people living with HIV/AIDS”	0.4 (0.3, 0.6)	0.3 (0.3, 0.5)	0.3 (0.3, 0.4)	0.3 (0.1, 1.0)
Q2 “I will know whether I am taking a sugar pill”	1.0 (0.9, 1.2)	1.1 (0.9, 1.2)	1.1 (0.9, 1.3)	2.0 (0.8, 4.9)
Q3 “This study will last for 3 years”	0.6 (0.4, 0.9)	0.7 (0.4, 1.0)	0.8 (0.5, 1.4)	∞
Q4 “Ten teaspoons of blood will be taken from my arm”	0.9 (0.7, 1.0)	1.0 (0.8, 1.2)	1.1 (0.9, 1.4)	2.7 (0.8, 9.0)
Q5 “I will have a chest x-ray done”	0.1 (0.04, 0.2)	0.1 (0.02, 0.1)	0.1 (0.02, 0.2)	0.1 (0.01, 0.8)
Q6 “I must continue taking the medicine even if the medication makes me ill”	0.9 (0.8, 1.0)	1.5 (1.2, 1.7)	1.8 (1.5, 2.3)	2.0 (0.7, 5.7)
Q7 “I will no longer receive study medication if I stop taking the medication”	0.7 (0.6, 0.8)	1.0 (0.9, 1.2)	1.4 (1.2, 1.6)	0.9 (0.4, 1.9)
Q8 “There is almost no chance that someone will find out I am HIV infected”	0.7 (0.6, 0.9)	0.7 (0.6, 0.9)	0.8 (0.6, 1.1)	0.9 (0.3, 3.0)
Q9 “By taking the medicine I have a very small chance of having damage to my liver”	0.5 (0.4, 0.6)	0.6 (0.5, 0.7)	0.6 (0.5, 0.8)	0.7 (0.3, 1.5)
Q10 “I will be seen by a doctor or nurse each time I return for a visit”	0.6 (0.4, 0.9)	0.7 (0.4, 1.0)	0.9 (0.5, 1.5)	∞
Q11 “I will not receive compensation for transport costs to come to the clinic”	1.3 (1.0, 1.7)	1.4 (1.1, 1.9)	1.8 (1.3, 2.5)	∞
Q12 “I can refuse to continue in the study at any time”	0.6 (0.5, 0.7)	0.9 (0.8, 1.1)	1.2 (0.9, 1.4)	0.9 (0.4, 2.1)
Q13 “I must give a urine sample at each visit”	3.4 (2.6, 4.4)	4.3 (3.2, 5.7)	3.8 (2.7, 5.2)	2.8 (0.7, 11.7)
Q14 “My blood will be tested for possible liver damage by the study medication”	1.7 (1.3, 2.3)	2.7 (1.9, 3.8)	3.6 (2.2, 5.7)	∞
Q15 “If I become ill with AIDS I will no longer be in the study”	2.5 (2.1, 3.1)	2.7 (2.2, 3.3)	3.6 (2.7, 4.8)	8.0 (1.1, 59.0)
Q16 “The isoniazid may make me sick by making me feel nauseated”	0.3 (0.2, 0.3)	0.4 (0.3, 0.4)	0.4 (0.3, 0.5)	0.4 (0.2, 1.1)
Q17 “Currently the Botswana government recommends one year of isoniazid preventive treatment for people living with HIV/AIDS”	0.7 (0.6, 0.8)	0.7 (0.6, 0.9)	0.8 (0.6, 1.0)	0.9 (0.3, 2.7)
Q18 “A placebo is a sugar pill”	0.4 (0.3, 0.5)	0.5 (0.4, 0.5)	0.5 (0.4, 0.6)	0.5 (0.2, 1.1)
Q19 “If I think I am hurt by this study I should contact the police station in Gaborone”	0.8 (0.6, 1.0)	1.0 (0.7, 1.3)	0.9 (0.6, 1.2)	∞
Q20 “This study will help people living with HIV/AIDS by finding out if longer preventive treatment with isoniazid will prevent TB”	0.3 (0.2, 0.5)	0.3 (0.2, 0.4)	0.2 (0.1, 0.3)	0.3 (0.1, 1.1)

## Discussion

This study measured comprehension among 1,835 adults in Botswana recruited for a longitudinal TB prevention trial through repeated administration of a quiz to test participants' comprehension of key study concepts. Participants who had a higher education or took the quizzes in English rather than Setswana demonstrated better comprehension. We also observed that repetition of the quiz during the 2-year period improved comprehension and that a significant proportion of participants had difficulty responding correctly to questions about placebo, randomization, and adverse events.

Having had more years of education was associated with higher rates of passing, both at enrollment and during follow-up. These data reinforce findings of many other studies that higher levels of education are associated with higher levels of understanding of medical research [10], [44]–[47]. Although the mother tongue of over 90% of the participants was Setswana (data not shown), preferring to take the quiz in English was associated with higher rates of passing at enrollment and follow-up. Individuals who took the quiz in English were more likely to pass regardless of highest level of education, and those with higher levels of education were more likely to pass regardless of the language in which the quiz was taken. There was also an association between language and educational attainment – those who chose to take the quiz in English had higher levels of education, on average, than those who chose to take the quiz in Setswana.

Examination of specific quiz items revealed that participants tended to answer questions correctly that related to study purpose and procedures, while some questions related to randomization, placebos, risks, and voluntariness were answered correctly less than 70% of the time. While it is troubling that these particular issues were problematic with respect to informed consent, it is consistent with literature demonstrating that these items are particularly likely to be misunderstood among subjects in both the developed and developing world [1], [3]–[5], [20].

Various investigators have tested mechanisms to improve how study information is delivered to participants with mixed results [20], [48]–[52]. Consent interventions that include more discussion seem to be most effective in reducing misunderstanding of study information [45], [53]–[55]. With regard to randomization in particular, there is evidence to suggest that providing explanations as to *why* subjects are being randomized (and not just how) improves subjects' understanding [8].

Quiz 2 was administered at 6 months after enrolment to a minority of participants: the range in the time since the enrolment quiz when quiz 2 was administered was broad. This heterogeneous timing of quiz 2's administration combined scores of participants who had taken the quiz 6 months previously with some who had received it years before and may have forgotten certain elements. In addition to this limitation, the observation that repeated quizzes improved comprehension may represent survivor bias: those who understood the study requirements may have been more likely to remain enrolled than those who did not. A further word of caution is that since there were only 20 comprehension questions, missing just two questions resulted in a 10% lower score, and although such a drop may have been determined to be a statistically significant decline, it may have not been meaningful with respect to actual comprehension. Future work might determine which subsets of quiz questions were considered by investigators to be particularly critical to participant understanding and examine trends in correctly answering these questions over time.

Back-translation of several items from the Setswana version of the quiz revealed discrepancies between the Setswana and English versions. While we adjusted what counted as a correct answer to correspond to the accurate meaning of one particular question, this likely introduced some confusion, particularly for the 53% who took the quiz in different languages on different study visits. Confusion may have been most acute for the items related to placebo. Specifically, 5 of 8 of our post-study translators reported that the phrase “sugar pill” from the Setswana quiz could be understood colloquially as “diabetes pill” because, according to the translators, in vernacular Setswana the term for “sugar” and “diabetes” is medically the same [56]. Translators further suggested that the meaning of the phrase is influenced by context; as the study was not about diabetes but TB prevention it should have been evident to the participant. That >25% of those who took the quiz in English responded incorrectly to quiz items related to placebos suggests that placebo remains a difficult concept to understand, regardless of language [5], [57], [58].

This discrepancy highlights the challenge of creating consent documents and other study materials in multiple languages. Presenting study information clearly is difficult in one's native language; accurately translating what are sometimes abstract or unusual concepts (such as randomization) has been consistently documented as problematic in international research [59]–[64].

True/false quizzes are an imperfect means of measuring understanding, as they allow participants to guess answers as well as to repeat back what they heard, whether or not they understood it. Research has demonstrated that asking participants to voice, in their own words, their understanding of research more thoroughly reveals what subjects understand [48], [65]. At the same time, the current standard is for no comprehension assessment to be conducted whatsoever, despite understanding being one of the four core elements of valid informed consent [66]. As such, conducting any type of assessment, including a quiz, is a significant step toward honoring this core tenet of consent.

### Conclusion

This study demonstrated that participants in a large 3-year clinical trial in Africa generally understood key study information. Importantly, it also demonstrated that administration of a quiz both at enrollment and follow-up was feasible and served as a useful means of determining whether subjects had sufficient information to enroll in the trial. Participants' understanding of information decreased slightly following enrollment, but the rate of passing improved following the first re-assessment, suggesting that providing quizzes over the course of an ongoing clinical trial may reinforce key study information. Assessments of understanding should be incorporated into future research, particularly in relation to topics that may be less familiar to research participants.
